# Factor XIII-A transglutaminase deficient mice show signs of metabolically healthy obesity on high fat diet

**DOI:** 10.1038/srep35574

**Published:** 2016-10-19

**Authors:** Vamsee D. Myneni, Aisha Mousa, Mari T. Kaartinen

**Affiliations:** 1 Faculty of Dentistry, McGill University, Montreal, QC, Canada; 2 Division of Experimental Medicine, Department of Medicine, Faculty of Medicine, McGill University, Montreal, QC, Canada.

## Abstract

*F13A1* gene, which encodes for Factor XIII-A blood clotting factor and a transglutaminase enzyme, was recently identified as a potential causative gene for obesity in humans. In our previous *in vitro* work, we showed that FXIII-A regulates preadipocyte differentiation and modulates insulin signaling via promoting plasma fibronectin assembly into the extracellular matrix. To understand the role of FXIII-A in whole body energy metabolism, here we have characterized the metabolic phenotype of *F13a1*−/− mice. *F13a1*−/− and *F13a1*+/+ type mice were fed chow or obesogenic, high fat diet for 20 weeks. Weight gain, total fat mass and fat pad mass, glucose handling, insulin sensitivity, energy expenditure and, morphological and biochemical analysis of adipose tissue was performed. We show that mice lacking FXIII-A gain weight on obesogenic diet, similarly as wild type mice, but exhibit a number of features of metabolically healthy obesity such as protection from developing diet-induced insulin resistance and hyperinsulinemia. Mice also show normal fasting glucose levels, larger adipocytes, decreased extracellular matrix accumulation and inflammation of adipose tissue, as well as decreased circulating triglycerides. This study reveals that FXIII-A transglutaminase can regulate whole body insulin sensitivity and may have a role in the development of diet-induced metabolic disturbances.

Obesity is a global epidemic and a major risk factor for the development of insulin resistance, type 2 diabetes, cardiovascular diseases, hypertension, respiratory diseases, osteoarthritis, and several types of cancers which lead to reduced life expectancy^1^,^2^,^3^,^4^, however, approximately 10–25% of obese humans remain healthy and are free of the metabolic consequences of weight gain^5^. This metabolically healthy obesity has been speculated to arise from protection to developing insulin resistance^5^ and altered adipose tissue properties in individuals^6^. Insulin resistance is the most common metabolic consequence of obesity characterized by hyperinsulinemia, hyperglycemia and dyslipidemia. Insulin resistance causes impaired insulin mediated glucose uptake in the muscle and adipose tissue, incomplete suppression of hepatic glucose output, and increase in lipolysis in adipose tissue, which leads to the storage of excess fat ectopically, which in turn further exacerbates insulin resistance and risks the development of type 2 diabetes^7^.

Many genome wide association studies have identified novel genes that contribute to the development of obesity and related metabolic disturbances as well as comorbidities^8^,^9^. Genome-wide association study using monozygotic twin pairs discordant in obesity, identified *F13A1* gene in white adipose tissue (WAT) as a potential causative gene for obesity. Furthermore, the GenMets cohort study identified seven small nucleotide polymorphisms (SNPs) in *F13A1* gene that were associated with body mass index (BMI)^10^ and one study linked *F13A1* SNP (rs7766109) with BMI and insulin resistance in polycystic ovary syndrome^11^. These studies suggest an important role for *F13A1* in energy metabolism.

*F13A1* encodes for Factor XIII-A enzyme (FXIII-A)(mouse gene; *F13a1*). FXIII-A is a blood-clotting factor and a transglutaminase enzyme^12^. The transglutaminase enzyme family is capable of modifying protein bound glutamine residues via transamidation reaction^12^,^13^,^14^. The best characterized modification is the formation of covalent N-glutamyl-ε-lysyl crosslinks, i.e., isopeptide bonds between substrate proteins^12^,^13^,^14^. In plasma, FXIII-A participates in clot stabilization during the hemostatic process where it creates a mechanically stronger clot by cross-linking fibrin chains and by incorporating antifibrinolytic proteins to it to prevent premature degradation of the clot by the fibrinolytic system^12^,^15^. Congenital FXIII-A deficiency, is a rare bleeding disorder that can present with recurrent miscarriages, poor wound healing, mucosal bleeding and life-threatening intracranial haemorrhage^12^,^16^. FXIII-A knockout mice display similar features seen in FXIII-A deficient patients, such as clotting defects, increased incidence of miscarriage and defective wound healing^17^. The circulating FXIII-A is predominantly produced by cells of bone marrow origin, such as megakaryocytes^18^, however, many other cell types such as monocytes^18^, macrophages^19^, chondrocytes^20^, osteoblasts, osteocytes^21^,^22^ and preadipocytes^23^ have now been reported to produce this enzyme. In these cell types FXIII-A can regulate cellular differentiation and extracellular matrix (ECM) deposition^12^,^13^,^14^. In our previous *in vitro* work, we demonstrated that FXIII-A produced by preadipocytes regulates 3T3-L1 preadipocyte differentiation and insulin sensitivity. FXIII-A transglutaminase activity promotes plasma fibronectin (FN) assembly to preadipocyte matrix, and the assembled FN matrix regulates adipocyte differentiation and insulin signalling^23^. In this study, we investigated the metabolic phenotype of FXIII-A null mice on obesogenic, high fat diet (HFD) and report that *F13a1*−/− mice exhibit number of features of metabolically healthy obesity such as protection to developing diet-induced insulin resistance, decreased ECM accumulation and reduced macrophage infiltration in WAT. Our study demonstrates for the first time in mice that that FXIII-A is involved in whole-body metabolic health.

## Materials and Methods

### Antibodies and reagents

Antibodies against rabbit anti-Akt (pan), rabbit anti- phospho-Akt (Ser^473^)(D9E), were purchased from Cell Signalling Technology Inc. (Beverly, MA, USA). Rabbit anti-fibronectin antibody was from EMD Millipore (Billerica, MA, USA). Mouse anti-fibronectin (EP5) purchased from Santa Cruz Biotechnology (Santa Cruz, CA, USA). Horseradish peroxidase-conjugated anti-mouse, and anti-rabbit IgG were from Jackson ImmunoResearch Inc. (West Grove, PA, USA). Glucose was measured by using The One Touch UltraMini glucometer (LIfeScan, Burnaby, BC, Canada). Novo rapid insulin was purchased from Novo Nordisk Canada (Mississauga, ON, Canada). All other reagents were purchased from Sigma or Fisher Scientific unless otherwise noted.

### Animals

*F13a1*−/− mice were kindly provided by Dr. Gerhardt Dickneite (Aventis Behring GMBH, Germany)^24^. Wild type mice (129Ola/CBA) obtained from Jackson laboratories and used for breeding. *F13a1*−/− and *F13a1*+/+ littermate control are used for this study. All mice are maintained in 12 h light/12-h dark cycle. Mice have ad libitum access to water and food. At 4 weeks of age, male *F13a1*−/− and *F13a1*+/+ were fed with either chow or high fat diet (HFD) (containing 60% Kcal from fat) (Harlan Laboratories) until the mice were 20 weeks old. All the experiments methods involving mice were carried out in accordance with the approved guidelines and regulations by the animal care committee of McGill University.

### Glucose tolerance test (GTT) and insulin tolerance test (ITT)

GTT and ITT were done at 18 weeks on a chow or HFD, after 6 h of fasting. For glucose tolerance test, mice were given intraperitoneal injections of glucose (1 g/Kg body weight) and blood glucose level was measured using glucometer at 0, 15, 30, 60, 120 min after glucose injection. For insulin tolerance test, insulin (0.5 U/Kg body weight) was injected and blood glucose levels were measured at 0, 30, 60, 90, 120 min after insulin injection. Area under the curve was calculated as previously described^25^.

### *In vivo* insulin signalling

Mice were fasted for 6 h, and injected intraperitoneally with human insulin or saline at a dose of 2.5 U/kg body weight. Mice were sacrificed at 10 min postinjection and tissues were homogenized in extraction buffer. The extraction buffer contains 100 mM Tris (pH 7.4), 1% Triton X-100, 10 mM EDTA, 100 mM sodium fluoride, 2 mM phenylmethylsulfonyl fluoride, 5 mM sodium orthovanadate, and protease inhibitor cocktail. The protein lysate was analyzed by Western blotting. The blots were probed with anti-Phospho-Akt (S473) and anti-total-Akt. Densitometric analysis of the bands was done by using the NIH Image J program.

### Indirect calorimetric measurements

Indirect calorimetry measurements were done at Mouse Metabolic Phenotyping Platform facility at McGill University. Animals were individually housed in metabolic chambers maintained at 20 to 22 °C on a 12-h/12-h light-dark cycle with lights on at 0700. Metabolic parameters (oxygen consumption, carbon dioxide production, respiratory exchange ratio (RER), and locomotor activity and energy expenditure) were obtained continuously using a TSE system. Mice were provided with the standard chow or HFD diet and water ad libitum. Presented results contain data collected for a period of 5 days following 5 days of adaptation to the metabolic cages.

### Histology and immunohistochemistry

20-week old mice adipose tissue and liver was fixed in 10% Neutral buffered formalin (NBF) overnight at RT, embedded in paraffin, sections were stained with hematoxylin and eosin-stain. Images of adipose tissue were taken from the center or the caudal end of the fat pad. In the analyses of frequency distribution of the adipocytes both types of images were used. For quantification of adipocyte area and number, 5–6 fields per section were averaged and four mice per group were used. Image J software was used to measure adipocyte area, the percentage of adipocytes in each 100-μm^2^ area and the average adipocyte area (in μm^2^). Adipocyte size and frequency distribution were measured from four mice/genotype (>500 cells/genotype) as previously published^26^. Immunohistochemistry of epididymal fat pad for macrophages was done using F4/80 as a marker as previously described^23^.

### Collagen and fibronectin quantification

Tissue collagen content was determined by sircol assay and hydroxyproline assay, which were done using a previously described protocol with minor modifications^27^. Briefly, 100 mg of epididymal and inguinal fat pads were sonicated in CHAPS detergent buffer (50 mM Tris-HCl, pH 7.4, 150 mM NaCl, 10 mM CHAPS, 3 mM EDTA and protease inhibitors). One hundred (100) μl of lysate was used for sircol and hydroxyproline assay. One hundred (100) mg of epididymal and inguinal fat pads were used to extract DOC-soluble and DOC-insoluble fractions and analyzed using ELISA as previously described^23^.

### Real time PCR

mRNA was isolated using Trizol method. Real-time PCR was performed on a ABIHT7900 RT-PCR machine using the comparative C_T_ method in triplicate using the TaqMan Universal Master Mix II. Expression levels of *F13a1* (Mm 00472334_m1), *Mcp-1* (Mm 00441242_m1), *Fn1* (Mm01256744_m1), *Col1a1* (Mm00801666_g1) was assessed and normalized to *Rn18S* (Mm 03928990_g1) or Gapdh (Mm99999915_g1).

### Insulin ELISA and Triglyceride assay

Insulin levels in plasma are measured using Ultra Sensitive Mouse Insulin ELISA Kit (Crystal Chem). Triglyceride levels in plasma were analyzed using triglyceride colorimetric assay kit (Cayman Chemical).

### Statistical analysis

Data are the standard error of the mean. Differences between groups were determined by ANOVA followed by Turkey’s post hoc tests or Student’s t-test as appropriate; p < 0.05 was considered significant.

## Results

### FXIII-A deficient mice show alterations in fat mass on chow and HFD

**Figure 1:**
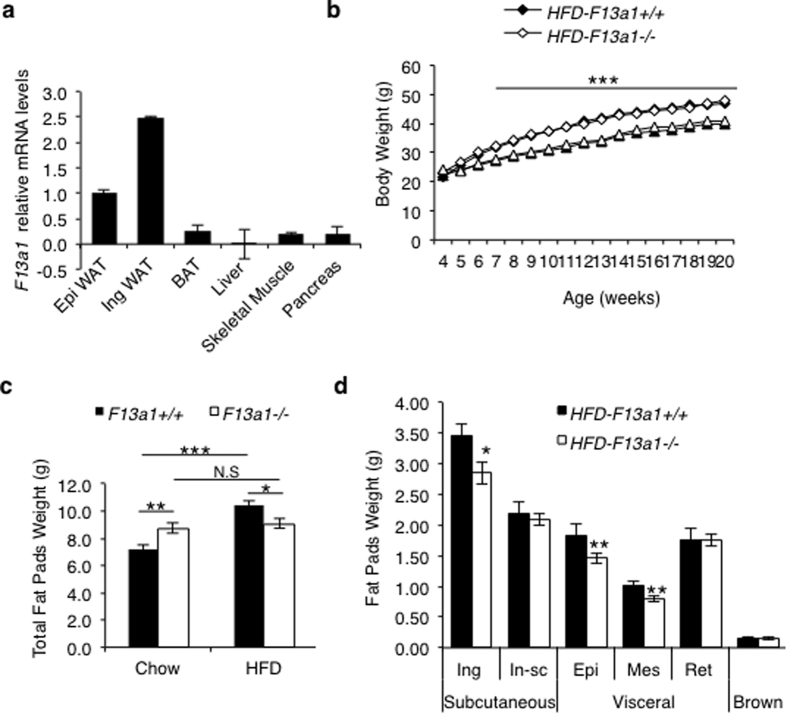
*F13a1*−/− mice on HFD are resistant to fat accumulation (**a**) *F13a1* mRNA expression in 1 month old mouse metabolic tissues; Epididymal (Epi WAT), Inguinal (Ing WAT); Brown adipose tissue (BAT), liver, skeletal muscle and pancreas (n = 3); Error bars represent SD **(b)** Body weights of *F13a1*−/− and *F13a1*+/+ mice fed chow or HFD (n = 9–11 mice/group). Significant difference between chow and HFD was observed after 4 weeks on the diets, i.e., from 8 weeks age onwards, however, no difference in weight gain between *F13a1*−/− and *F13a1*+/+ mice were observed. **(c)** Total fat pad weights from *F13a1*−/− and *F13a1*+/+ mice on chow or HFD show that *F13a1*−/− mice do not add significant fat mass (data is collected from data presented in Figure S1b,d). **(d)** Weights of individual fat pads of mice on HFD. Subcutaneous fat: inguinal (Ing) and inter-scapular (In-sc); visceral: epididymal (Epi), mesenteric (Mes) and retroperitoneal (Ret) fat pads (n = 10–16 mice/group). Error bars represent SEM; *p < 0.05; **p < 0.01; ***p < 0.001; N.S.-Not Significant.

Analyses of *F13a1* mRNA levels in metabolic tissues that regulate whole body glucose and energy balance, i.e., in WAT (subcutaneous and visceral), brown adipose tissue (BAT), liver, skeletal muscle and pancreas, showed that *F13a1* mRNA levels are the highest in subcutaneous WAT depot (inguinal WAT) - levels were two and half-fold higher than in visceral WAT (epididymal WAT). Other metabolic tissues showed negligible expression (Fig. 1a).

To determine the potential role of *F13a1* gene on the development of obesity and its associated metabolic complications, 4-week old, male *F13a1*+/+ and *F13a1*−/− mice were fed either a normal diet (chow) or obesogenic, HFD until 20 weeks age. *F13a1*+/+ and *F13a1*−/− mice fed chow or HFD, showed similar growth and weight gain, i.e., F13a1−/− did not gain more or less weight than control mice on HFD (Fig. 1b). The food intake of *F13a1*+/+ and *F13a1*−/− mice fed on chow diet or HFD were unaltered (Supplementary Figure S1a,c). Surprisingly, even though *F13a1*−/− mice gained body weight on HFD, these mice showed resistance to fat accumulation in adipose tissue. While the *F13a1*+/+ mice gained 44% fat mass on HFD, FXIII-A deficient mice gained only 3.4% in fat mass (Fig. 1c). On HFD, *F13a1*−/− mice showed decreased fat accumulation specifically to epididymal (−20%), mesenteric (−21%), inguinal (−18%) fat pads as compared to *F13a1*+/+ mice (Fig. 1d). There was no redistribution of fat between visceral and subcutaneous fat depots in the *F13a1*−/− mice on both chow and HFD (Supplementary Figure S1d).

Interestingly, *F13a1*−/− on chow diet at 20-weeks showed a significant *increase* in the fat pad weights compared to *F13a1*+/+ mice showing that these mice are ‘fattier’ on a normal diet (Fig. 1c, Supplementary S1b). Lean mass on HFD was not significantly different in *F13a1*+/+ and *F13a1*−/− mice (Supplementary S1e). Changes in fat mass was not because of increased physical activity and/or energy consumption, as evidenced by energy parameters from metabolic chamber studies. Surprisingly, physical activity was mildly, but significantly reduced in *F13a1*−/− mice compared to *F13a1*+/+ mice on HFD (Supplementary Figure S2a). *F13a1*−/− mice also showed reduced energy expenditure (EE), reduced oxygen consumption (VO2) and carbon dioxide production (VCO2) (Supplementary Figure S2) compared to *F13a1*+/+ mice on HFD. The respiratory exchange ratio (RER) was not altered on chow or HFD (Supplementary Figure S2e). Circulating triglyceride levels (Supplementary Figure S3) were significantly lower in *F13a1*−/− mice compared to *F13a1*+/+ mice on HFD but no differences in liver triglyceride levels were observed (data not shown).

### FXIII-A deficiency protects mice from diet-induced insulin resistance

**Figure 2:**
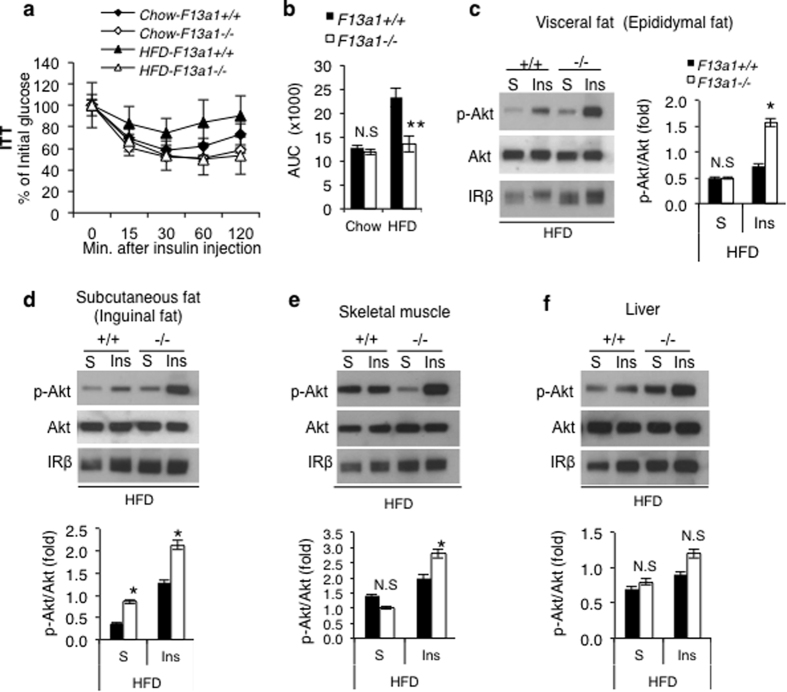
Enhanced insulin sensitivity in *F13a1*−/− mice fed on HFD. (**a)** Insulin tolerance test (ITT) shows that *F13a1*−/− mice on HFD have similar insulin sensitivity as on chow, where as *F13a1*+/+ show insulin resistance on HFD. **(b)** AUC for ITT shows a significant protection from insulin resistance in *F13a1*−/− mice on HFD (n = 9–11 mice/group) **(c–f)** *In vivo* insulin signalling in metabolic tissues (epididymal fat, inguinal fat, skeletal muscle and liver) of *F13a1*−/− and *F13a1*+/+ mice. Mice were fasted for 6 h, and injected with saline (S) or insulin (Ins) (2.5 U/kg). Tissues were dissected immediately and total protein extracts were prepared and analyzed by Western blotting for p-Akt (S473), total Akt levels and insulin receptor (IRβ). All error bars represent SEM; (n = 3 mice/group); *p < 0.05; **p < 0.01; N.S.-Not Significant.

To further explore the effects of FXIII-A deficiency on energy metabolism, metabolic parameters in *F13a1*−/− and *F13a1*+/+ mice were analyzed. The most commonly known metabolic consequence of obesity is insulin resistance^28^. Insulin tolerance test (ITT) of *F13a1*−/− and *F13a1*+/+ mice on chow diet showed that mice have similar insulin sensitivity, however, while *F13a1*+/+ mice develop insulin resistance on HFD, *F13a1*−/− mice were protected and showed normal insulin sensitivity (as on chow diet) (Fig. 2a,b). To further explore the systemic insulin sensitivity of *F13a1*−/− mice on HFD, insulin signalling in peripheral tissues, i.e., WAT, skeletal muscle and liver was assessed. At 20 weeks of age, mice were injected with insulin, peripheral metabolic tissues were immediately dissected, and total protein extracts were analyzed for Akt phosphorylation by Western blotting. In *F13a1*−/− mice, insulin induced a significantly robust increase in Akt Ser-473 phosphorylation without affecting total insulin receptor levels (IRβ) (Fig. 2c–f). This increase in insulin sensitivity in HFD-fed *F13a1*−/− mice were observed in the epididymal fat pad (Fig. 2c), inguinal fat pad (Fig. 2d) and skeletal muscle (Fig. 2e), but not in liver (Fig. 2f).

**Figure 3:**
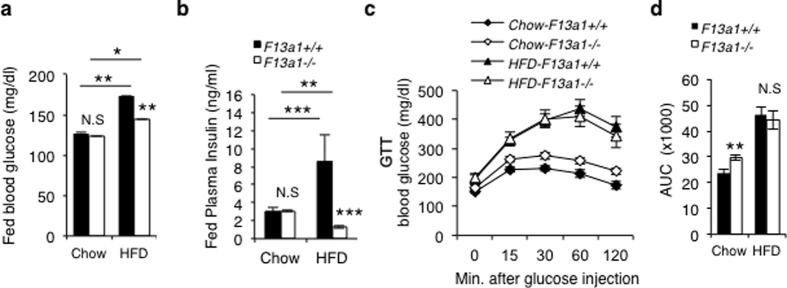
Glucose metabolism in *F13a1*−/− *and F13a1*+/+ mice. **(a)** Analysis of fed state blood glucose levels shows significantly lower blood glucose in *F13a1*−/− mice compared to controls whose blood glucose is raised on HFD. (**b**) Fed state plasma insulin levels on chow and HFD, *F13a1*−/− mice on HFD show a significantly decreased insulin levels compared to *F13a1*+/+ mice. **(c,d)** Glucose Tolerance Test (GTT) and GTT AUC (area under the curve) shows that F13a1−/− do develop glucose intolerance on HFD; (n = 9–11 mice/group) *p < 0.05; **p < 0.01; N.S.-Not Significant.

Random blood glucose levels in *F13a1*−/− mice on HFD was 17% lower in comparison to *F13a1*+/+ mice (Fig. 3a). Fasting blood glucose levels on chow or HFD showed no significant differences (Supplementary Figure S4). Plasma insulin levels of HFD-fed *F13a1*−/− mice were significantly lower compared to *F13a1*+/+ mice (Fig. 3b) – demonstrating that F13a1−/− mice are resistant to developing hyperinsulinemia, which often accompanies insulin resistance. However, actual glucose tolerance test (GTT) showed that both *F13a1*−/− and *F13a1*+/+ mice develop impaired glucose clearance on HFD and show signs of prediabetes (Fig. 3c,d). Interestingly, the *F13a1*−/− mice on chow diet showed significantly impaired glucose clearance compared to *F13a1*+/+ mice (Fig. 3c,d) which may suggest that FXIII-A might have a role in pancreatic function in normal dietary conditions. Overall these results suggest *F13a1*−/− mice are protected from high fat induced insulin resistance.

### Large adipocytes, increased cell proliferation and alterations in ECM accumulation in HFD-fed FXIII-A deficient mouse WAT

**Figure 4:**
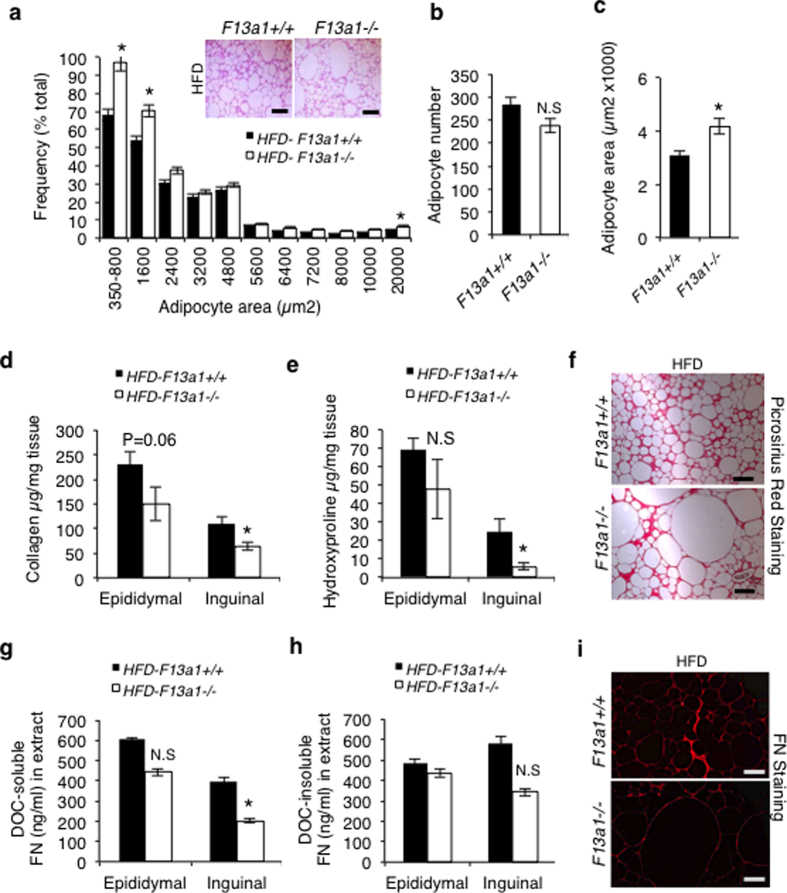
Larger adipocytes and decreased extracellular matrix accumulation in WAT of *F13a1*−/− mice fed HFD. (**a)** Frequency distribution of adipocytes in *F13a1*−/− and *F13a1*+/+ mice on HFD diet; the frequency of small and larger adipocytes were increased in *F13a1*−/− mice. Inset-Hematoxylin and Eosin (H&E) stained sections of epididymal fat pads show larger adipocytes. **(b)** The total adipocyte number was not increased. **(c)** An average adipocyte area with image of largest adipocytes found in histology shows significant increase in size. 5–6 fields per mice were counted. **(d,e)** Sircol and hydroxyproline assay for the collagen content of total protein extracts of epididymal and inguinal fat pads shows a significant decrease in inguinal fat pads. **(f)** Picrosirus red staining for collagen in epididymal fat pad. **(g)** (Deoxycholic acid) DOC-soluble and DOC-insoluble FN levels in epididymal and inguinal fat pads show a decrease in inguinal depot. **(h)** FN levels in DOC-insoluble fraction. **(i)** Immunofluorescence staining of FN in epididymal fat pad. All error bars represent SEM; Scale bar equals 1000 μm; (n = 4 mice/group); *p < 0.05; N.S.-Not Significant.

Improved insulin sensitivity has been shown to be associated with smaller adipocyte size^29^,^30^ or in some cases with larger adipocyte size as a sign of improved and less restricted adipocyte expansion^28^,^31^. The amount of visceral fat best correlates with insulin sensitivity in both animal models and humans, and visceral fat can account for most of the variability in insulin sensitivity in a heterogeneous population^32^, thus it was chosen for the following analyses. Histological examination and frequency distribution analysis of adipocytes in the visceral fat. i.e., epidydimal fat pad on chow diet showed a shift in profile of larger adipocytes in *F13a1*−/− mice (Supplementary Figure S5a,b). On HFD, the *F13a1*−/− mice show a bimodal distribution of both small and very large adipocytes compared to *F13a1*+/+ mice at 20 weeks of age (Fig. 4a). The total number of adipocytes was not altered (Fig. 4b), but the average adipocyte area is dramatically increased in HFD-fed *F13a1*−/− mice (Fig. 4c). Assessment of cell proliferation with Ki67 staining showed significant increase in proliferating cells in *F13a1*−/− mice supporting the idea of the increased amount of smaller cells (Supplementary Figure S6). The apparent contradiction between increased proliferation and unaltered adipocyte number in these mice can be explained by the fact that only mature adipocytes, that do not proliferate, can be counted and that proliferating cells may represent other cell types.

The presence of larger adipocytes in *F13a1*−/− mice may suggest a less restrictive ECM which might also contribute to the improved insulin sensitivity. Analysis of total collagen levels in epididymal and inguinal fat pads using sircol and hydroxyproline assays showed a significant decrease in collagen levels in *F13a1*−/− mice in inguinal fat pads, but not in epididymal fat pads compared to *F13a1*+/+ mice on HFD (Fig. 4d–f). Our previous work demonstrated that *F13a1*−/− MEFs display reduced FN assembly during adipocyte differentiation^23^. Analysis of FN levels in the DOC-soluble and DOC-insoluble tissue extracts show that in inguinal fat pad, the DOC-soluble FN levels were significantly reduced (Fig. 4g). FN in the DOC-insoluble fraction was decreased, but not significantly in *F13a1*−/− mice compared to *F13a1*+/+ mice on HFD (Fig. 4h). Epididymal fat pads showed no significant differences (Fig. 4g,h). WAT histology for FN showed a visible decrease in FN level in *F13a1*−/− mice on HFD compared to *F13a1*+/+ mice (Fig. 4i). mRNA expression of *Fn1* and *Col1a1* were increased on HFD in both epididymal and inguinal fat pads of *F13a1*+/+ and *F13a1*−/− mice (Supplementary Figure S7a–d), as expected^31^,^33^. Comparing the mRNA expression of *Fn1* and *Col1a1* of *F13a1*+/+ and *F13a1*−/− mice showed that, *Fn* mRNA levels were not significantly different in epididymal fat pad (Supplementary Figure S7e), but was increased in inguinal fat (Supplementary Figure S7g). *Col1a1* expression was decreased in epididymal fat (Supplementary Figure S7f), but no significant changes were seen in inguinal fat pad (Supplementary Figure S7h). The increase in *Fn* mRNA in inguinal fat does not correlate with the observed protein accumulation levels, which were decreased. This may suggest that the effect on mRNA expression is due to compensation from lack of FN matrix. The decrease in *Col1a1* levels may contribute to the observed lower hydroxyproline levels in the epididymal fat tissue, however, the COL I levels are significantly lower only in inguinal fat. This is because the effects of FXIII-A on COL I matrix is at the ECM and not at the translational level and that mRNA changes may be due to indirect and compensatory effects. Skeletal muscle of *F13a1*−/− mice also showed improved insulin sensitivity, and studies have shown that insulin resistance in muscle was associated with the increased hydroxyproline content in type 2 diabetic patients^34^. However, hydroxyproline assays of skeletal muscle show no difference between *F13a1*−/− and *F13a1*+/+ mice on HFD (Supplementary Figure S8).

### FXIII-A deficient mice on HFD show decreased adipose tissue inflammation

**Figure 5:**
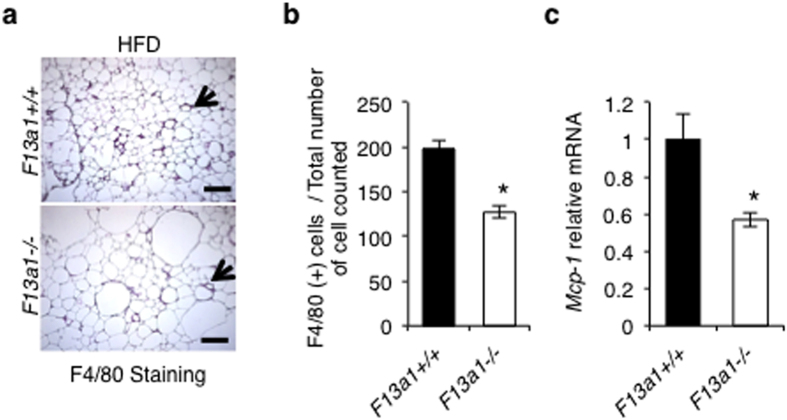
Reduced-macrophage content in WAT and decreased ectopic lipid accumulation in *F13a1*−/− mice on HFD. **(a)** Immunohistochemistry for F4/80-positive cells in the epididymal fat pad from *F13a1*−/− and *F13a1*+/+ mice on HFD. Arrows point to crown-like structures caused by macrophages. **(b)** Quantification of F4/80-positive cells from stained tissue sections. The total number of cells counted equal the total number of adipocytes plus F4/80 # per field **(c**) mRNA levels of macrophage marker *Mcp-1* gene in the epididymal fat pads. All error bars represent SEM; Scale bar equals 1000 μm; (n = 4 mice/group); *p < 0.05.

WAT inflammation and thus the macrophage number in adipose tissue is increased in obesity, and this is linked to development of diet-induced insulin resistance^35^. FXIII-A has been shown to express in macrophages and to decrease their migration^36^,^37^. Immunohistochemical staining for F4/80 of WAT showed localized macrophages in the crown-like structures surrounding adipocytes (Fig. 5a); quantification show that the number of crown-like structures are significantly lower in *F13a1*−/− mice compared to *F13a1*+/+ mice on HFD (Fig. 5b). The mRNA expression levels of the macrophage marker *Mcp-1,* which is best marker for macrophage accumulation in white adipose tissue and obesity induced insulin resistance^38^, is also significantly decreased in *F13a1*−/− mice WAT (Fig. 5c) suggesting decreased WAT inflammation.

## Discussion

*F13A1* gene, which encodes for FXIII-A transglutaminase, was recently identified as a potentially causative gene for obesity in human WAT, and several SNPs in *F13A1* gene were reported to associate with BMI^10^ and insulin resistance^11^. The role of FXIII-A in the development of obesity and/or its metabolic consequences has not been established. Here we provide first evidence that FXIII-A can regulate metabolic health in obesity. Global elimination of *F13a1* reduced WAT mass on HFD, although, *F13a1*−/− mice on HFD gained the same body weight as their wild type controls. *F13a1*−/− mice were also resistant to developing diet-induced insulin resistance and hyperinsulinemia and exhibited characteristics of metabolically healthy obesity, which include signs of healthy adipose tissue expansion such as an increase in adipocyte size, increase in proliferation, decrease in ECM content of subcutaneous fat, reduced WAT inflammation, and lower circulating triglyceride levels. In conclusion, the global absence of FXIII-A appears to be protective of some obesity-linked metabolic disturbances.

While some of the metabolic phenotype in *F13a1*−/− is clear, there are few observations that are perplexing and challenging to interpret. First is the observation that *F13a1*−/− mice gain weight but not fat mass or lean mass considering that energy consumption in these mice is not increased. It is possible that *F13a1*−/− mice have an altered fat absorption as FXIII-A was reported to play a significant role in the intestines and contributes to Crohn’s disease and ulcerative colitis^39^. However, the fact that mice are gaining weight supports the idea that lipids are indeed used for energy to increase the weight of other tissues. The lean mass quantification more accurately describes the muscle mass equlance, and no value for other tissues that contribute to weight, such as bone (bone organic matrix and mineral phase) is obtained from these measurements^40^. In fact, it is known that insulin has an anabolic effect on bone^41^ and our work has show a role for FXIII-A in bone and bone cells^21^,^22^,^42^,^43^. We are currently exploring the bone mass accrual and insulin sensitivity of *F13a1* deficient bone cells and the bone phenotype of the chow and HFD fed *F13a1*−/− mice. Another perplexing observation is that despite the improved insulin sensitivity of WAT, and larger adipocyte size in WAT, *F13a1*−/− mice showed decreased lipid accumulation in adipose tissue on HFD. These two phenomena do seem contradictory, however, this may be due to increased lipid mobilization which can occur in case of altered adipocyte cytoskeletal remodeling, which disrupts lipid maintenance and dynamics in adipose tissue^28^,^44^. In other words, the increased adipocyte size, in fat pads may indicate that the *F13a1* deficient adipocytes are accumulating fat easier, but does not retain the fat and are prone to increased lipid mobilization. The WAT cellular turnover and remodeling may also be increased and contribute to these events causing a net loss of fat mass but larger adipocytes. Indeed, ECM of WAT has been linked to lipid retention and WAT cellular turnover^33^,^28^,^45^.

A number of ECM molecules are transglutaminase and/or FXIII-A substrates, i.e., these proteins are crosslinked and stabilized by transglutaminases. Substrates include osteopontin which is required for fat accumulation^46^,^47^. Furthermore, osteopontin null mice showed similar metabolic and adipose tissue phenotype, i.e., protection from developing insulin resistance, reduced inflammation of WAT, and reduced collagen content in WAT, however, in comparison to FXIII-A null mice, osteopontin deficient mice did not gain weight on HFD and showed no adipocyte hypertrophy^48^,^49^,^50^. It remains to be seen if osteopontin acts as a substrate of FXIII-A in WAT and if FXIII-A regulates osteopontin function. However, it is clear that ECM components are involved in the development of metabolic disturbances, and they appear to have varied effects on adipocyte biology and some of which may thus add to the phenotype of *F13a1* null mice. WAT ECM mainly consists of collagens^33^ which we show here to be reduced in *F13a1*−/− WAT (at protein level). Lower collagenous WAT content may be a major contributor to the healthy metabolic phenotype. It has been shown that knocking out collagen type VI in *ob/ob* mice resulted in an improved metabolic profile and improved insulin sensitivity, which is attributed to the enhanced WAT expansion and the increase in adipocyte cell size^31^. Indeed, we also observed an increase in size of adipocytes and reduced collagen levels in WAT of *F13a1*−/− mice (in subcutaneous fat depot), and also observed both smaller adipocytes along with very large adipocytes in *F13a1*−/− deficient WAT. The presence of both small and large cells was also reported in mice treated with PPARγ agonist, which also caused improved insulin sensitivity^51^. It is plausable that also the observed increased cell proliferation in *F13a1*−/− WAT is linked to decreased ECM levels as it has been reported that increase in ECM deposition reduces the amount of adipocyte progenitors in WAT, contributing to loss of proper function and restricts adipocyte expansion via cellular proliferation (hyperplasia)^52^. The role of FXIII-A in ECM accumulation in WAT is also supported by the fact that although both control mice and *F13a1*−/− mice have a significant induction of FN and COL I mRNA on HFD, which is known to occur^31^,^33^. *F13a1*−/− mice nonetheless accumulate less ECM at protein level than control mice, suggesting that FXIII-A modulates ECM protein levels in the ECM space.

Collagen assembly in the ECM can be regulated by FN^53^. FN is one of the major extracellular transglutaminase substrate^54^ and a negative regulator of adipogenesis *in vitro,* where it inhibits lipid accumulation by blocking the morphological and cytoskeletal changes necessary for lipid accumulation^55^,^56^,^57^,^58^,^59^. FXIII-A has been linked to the ECM accumulation in many tissues and in cell culture models, particularly in the assembly of plasma FN, which is also its substrate in wound healing following blood clot formation^43^,^54^,^60^,^61^. In our previous study, we showed that FXIII-A is required for plasma FN assembly in 3T3-L1 preadipocytes and mouse embryonic fibroblasts cultures, the assembled plasma FN matrix promoted preadipocyte proliferation and inhibited differentiation. Indeed, we observed decreased FN levels, particularly in subcutaneous, inguinal WAT, where *F13a1* mRNA expression levels were the highest of the metabolic tissues tested. However, we did not observe a decrease in cell proliferation as would have been predicted from our *in vitro* studies. This may be because *in vivo* result likely arises from alterations in total ECM levels in WAT rather than decreased levels of plasma FN alone.

A major factor contributing to metabolic failure in obesity is WAT inflammation, characterized by increased levels of macrophages in the tissue. This macrophage infiltration into WAT forms crown-like structures (CLSs) which represent dying adipocytes^62^. The density of CLSs correlates with insulin resistance and proinflammatory environment^63^. *F13a1*−/− mice showed decreased levels of CLSs in WAT suggesting an improved inflammatory profile and healthy WAT. It is possible that this phenotype results from the combination of lack of FXIII-A in adipocytes, and lack of FXIII-A in macrophages themselves as FXIII-A in macrophages was shown to play a role in migration and phagocytosis^64^. Thus, in the absence of FXIII-A, macrophage function would be expected to be compromised which could contribute to reduced macrophage levels in WAT. Macrophage accumulation in WAT may also be linked to reduced levels of ECM or reduced crosslinking of ECM components as macrophages generally localized to profibrotic areas^65^.

In summary, we have found that mice deficient in FXIII-A show characteristics of metabolically healthy obesity with improved insulin sensitivity. Our results suggest that the absence of FXIII-A enzyme does not influence weight in a causative manner, but appears to modulate fat mass, improve insulin sensitivity and improve whole body metabolic health on HFD. Thus our work identified a novel role for FXIII-A in energy metabolism. Seven SNPs have been identified in *F13A1* gene in human WAT that link to BMI^10^. In the light of our findings, these SNPs may influence *F13A1* by modulating its expression in WAT which may increase or decrease the activity of the enzyme with various outcomes on metabolism. The observation that WAT and glucose metabolism are different in *F13a1*−/− mice on normal versus HFD diet suggests that the FXIII-A levels react to the needs of WAT and that its function may range from physiological to pathological depending how much of the enzyme is present in the tissue. V34L polymorphism in *F13A1* was shown to increase enzyme activation, but was not associated with obesity^12^,^66^ and no information is available on the metabolic health of FXIII-A deficient humans. Since it is not either known how obesity-linked, SNPs influence FXIII-A enzyme levels and/or activity, we cannot exclude the possibility that they may cause an increase in FXIII-A levels in WAT and the individual’s propensity to increase fat mass and to develop metabolic disturbances. In fact, based on this study, this could be predicted. Thus, FXIII-A may be be a valuable molecular target to improve the metabolic profile in obesity, and to regulate insulin resistance^24^.

## Additional Information

**How to cite this article**: Myneni, V. D. *et al.* Factor XIII-A transglutaminase deficient mice show signs of metabolically healthy obesity on high fat diet. *Sci. Rep.* **6**, 35574; doi: 10.1038/srep35574 (2016).

## Supplementary Material

Supplementary Information
