# Supplementary material for: Factor XIII-A transglutaminase deficient mice show signs of metabolically healthy obesity on high fat diet
Source: Sci Rep. 2016 Oct 19;6:35574. doi: 10.1038/srep35574 (PMC5069677; doi:10.1038/srep35574)
Supplement: Supplementary Information [file srep35574-s1.pdf]

Factor XIII-A transglutaminase deficient mice show signs of metabolically healthy obesity on high fat diet

Vamsee D. Myneni[1] and Mari T. Kaartinen[1,2]

[1]Faculty of Dentistry, McGill University, Montreal, QC, Canada,

[2]Division of Experimental Medicine, Department of Medicine, Faculty of Medicine, McGill University, Montreal, QC.

Supplemental Information
Figure S1

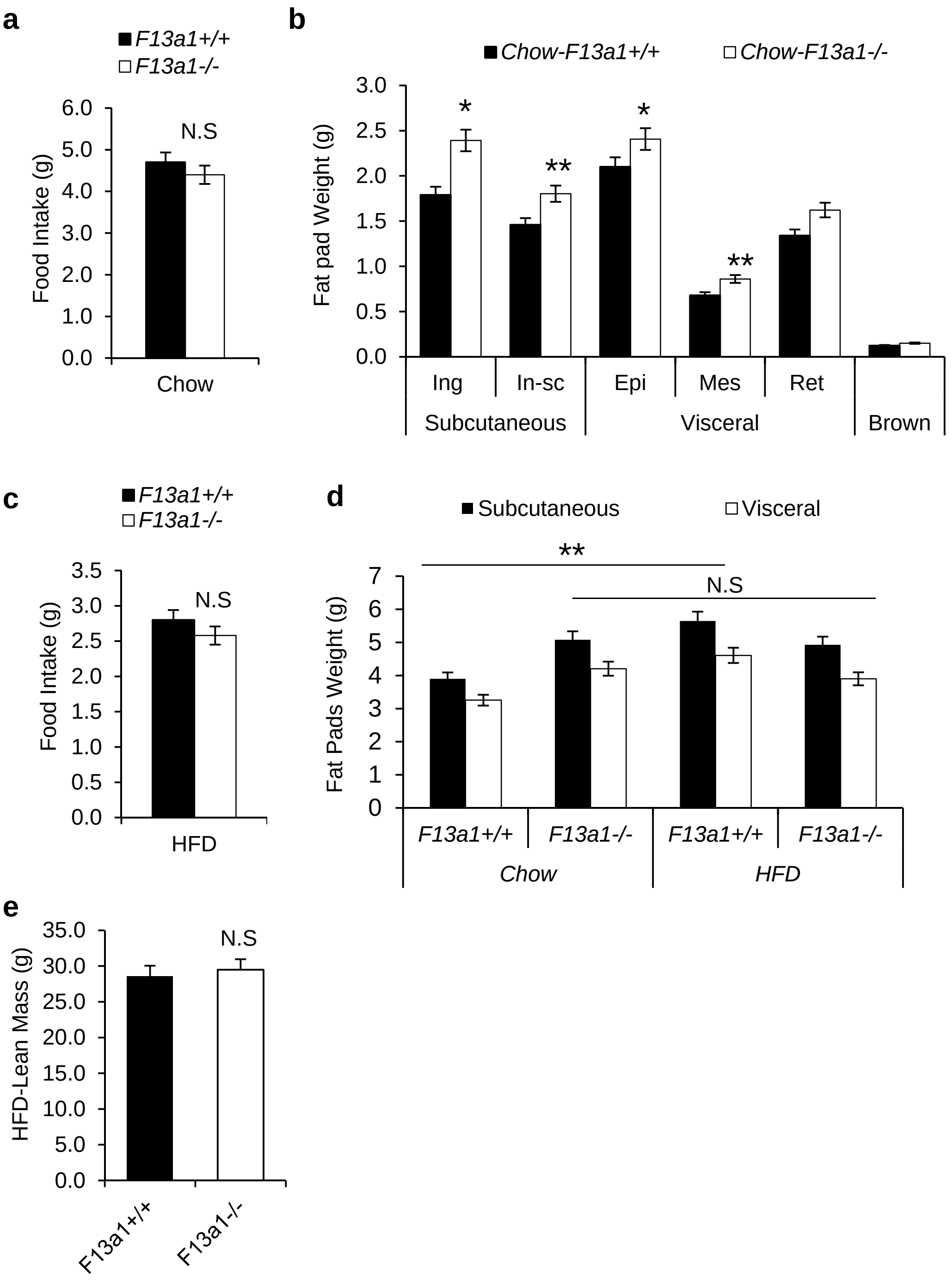

Supplemental Information
Figure S2

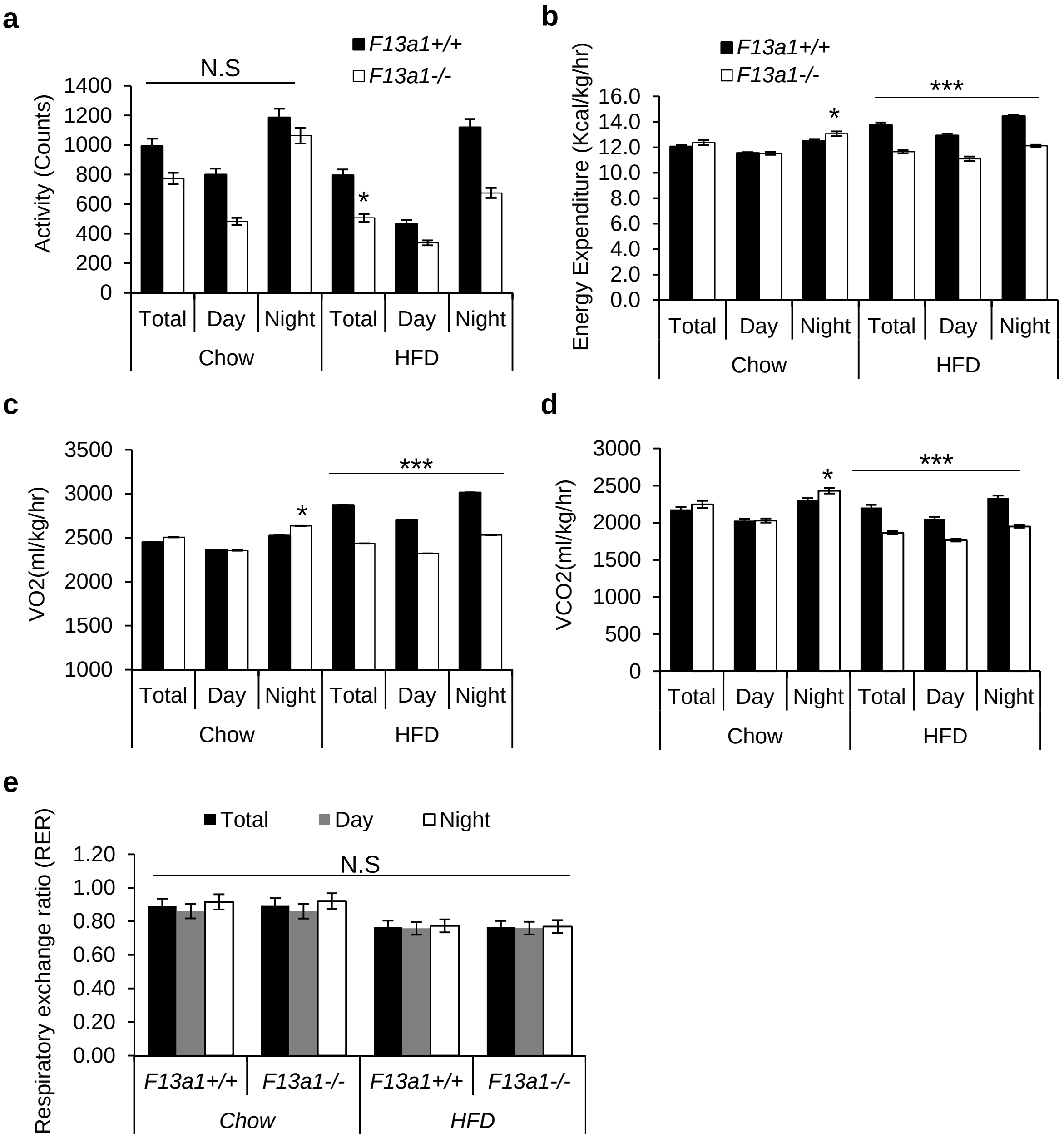

Supplemental Information
Figure S3

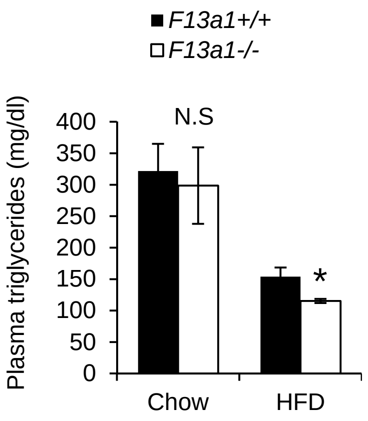

Supplemental Information
Figure S4

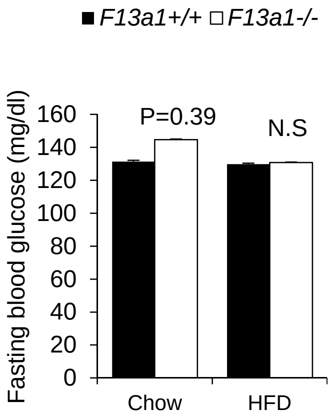

Supplemental Information
Figure S5

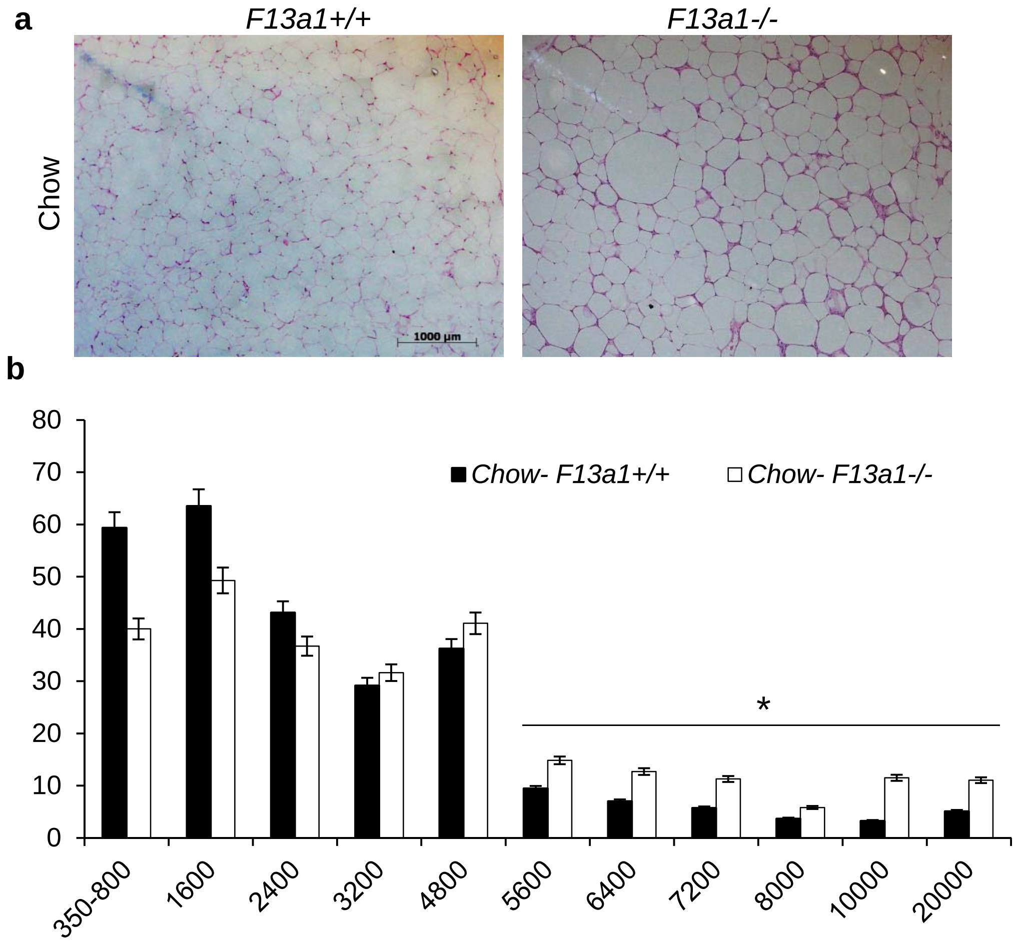

Supplemental Information
Figure S6

a

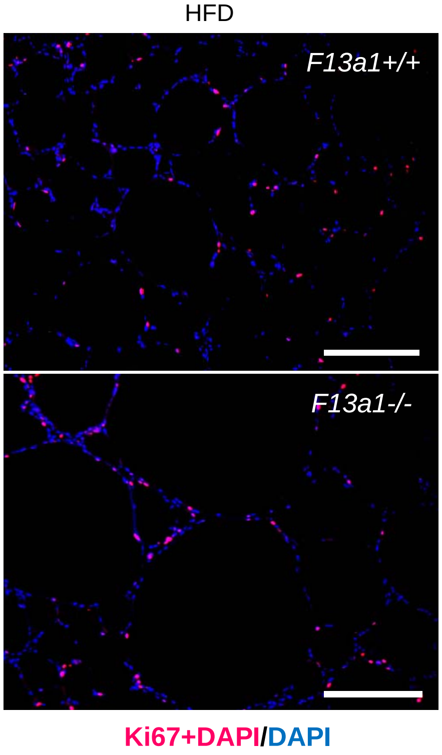

b

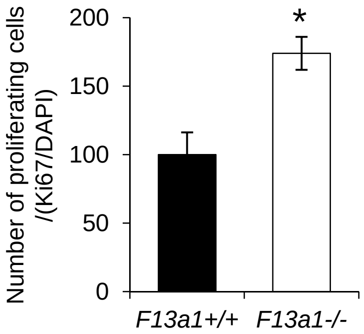

Supplemental Information
Figure S7

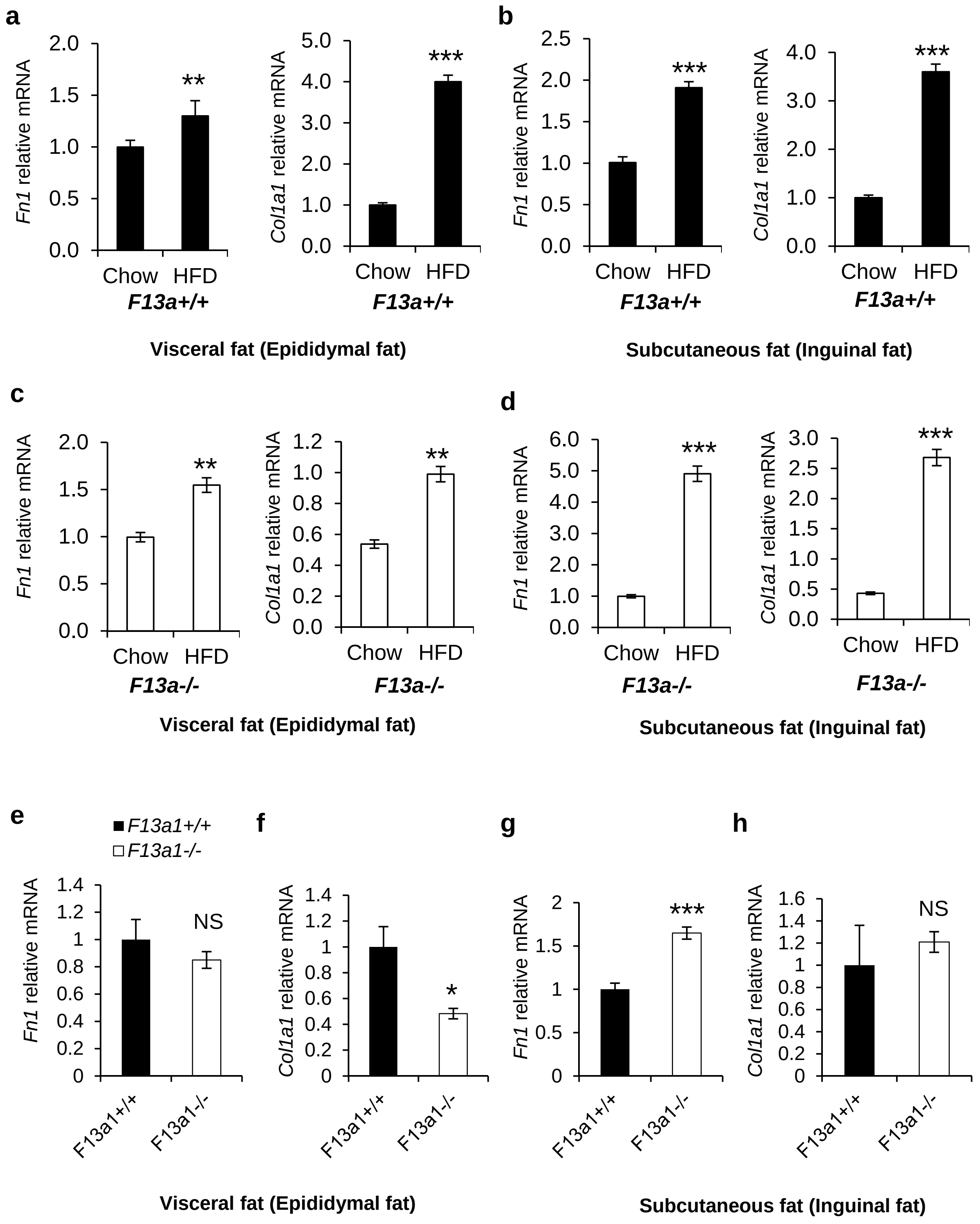

Supplemental Information
Figure S8

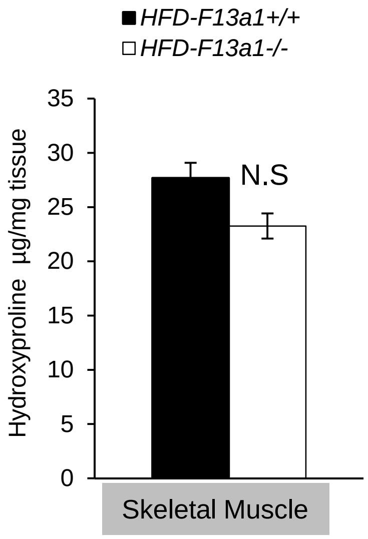

Factor XIII-A transglutaminase deficient mice show signs of metabolically healthy obesity on high fat diet

Vamsee D. Myneni and Mari T. Kaartinen

Supplementary figure legends

Figure S1. Increased fat pad weight in *F13a1-/-* mice fed on chow diet. (a) Food intake of *F13a1-/-* and *F13a1+/+* mice on chow diet (n=4 mice/group). **(b)** Individual fat pad's weight of *F13a1-/-* and *F13a1+/+* mice fed chow diet. Subcutaneous fat: inguinal (Ing) and inter-scapular (In-sc); Visceral: epididymal (Epi), mesenteric (Mes) and Retroperitoneal (Ret) fat pads; and brown fat (n=10-16 mice/group). **(c)** Food intake of *F13a1-/-* and *F13a1+/+* mice on HFD (n=4 mice/group). **(d)** Subcutaneous and visceral fat pad weight from *F13a1-/-* and *F13a1+/+* mice on chow and HFD; *F13a1-/-* mice do not show redistribution of fat mass on both chow and HDF (data is collected from Figures S1b and 1d). **(e)** HFD Lean Mass measured by EchoMRI (n=4 mice/group). No changes are observed. All error bars represent SEM; *p<0.05; **p<0.01; N.S-Not Significant.

Figure S2. Reduced activity and energy expenditure of *F13a1-/-* mice on HFD. Metabolic cage measurements were taken during the day (light) and night (dark) cycles over the course of four days. **(a)** Total activity (ambulatory and rearing) was reduced in *F13a1-/-* mice on HFD. **(b)** Energy expenditure was reduced in *F13a1-/-* mice on HFD**. (c)** Oxygen consumption (VO2) was significantly lower in *F13a1-/-* mice on HFD. **(d)** Carbon dioxide production (VCO2) was significantly lower in *F13a1-/-* mice on HFD. **(e)** Gas exchange data used to calculate the respiratory exchange ratio (RER=VO2/VCO2). All error bars represent SEM. (n=4 mice/group) *p<0.05; **p<0.01; ***p<0.001, N.S-Not Significant.

Figure S3. Plasma triglyceride levels on chow and HFD. Plasma triglyceride levels of *F13a1-/-* and *F13a1+/+* mice on HFD are significantly lower in *F13a1-/-* compared to control mice. All error bars represent SEM; (n=4 mice/group); *p<0.05, , N.S-Not Significant.

Figure S4. Fasting blood glucose levels are not affected. Blood glucose levels are measured after 6 h fast (7:00 am-1:00 pm). No significant differences are seen between *F13a1-/- and F13a1+/+* mice on chow or HFD. All error bars represent SEM. (n=8 mice/group) N.S-Not Significant.

Figure S5. Larger adipocytes in WAT of *F13a1-/-* mice on chow. (a) Hematoxylin and Eosin (H&E) stained sections of epididymal fat pad. Scale bar equals 1000 µm **(b)** Frequency distribution of adipocytes in mice on chow diet; the frequency of larger adipocytes was increased in *F13a1-/-* mice. Error bars represent SEM. (n=4 mice/group) *p<0.05.

Figure S6. Increased cell proliferation in WAT of *F13a1-/-* mice on HFD. (a) Immunofluroscence staining of the epididymal fat pad of *F13a1-/-* and *F13a1+/+* mice on HFD for Ki67 (red) and nuclei/DAPI (blue). Proliferating cells shown in pink nuclei (merge of Ki67 and DAPI). Scale bar equals 200 µm. **(b)** Graph represents quantification of proliferating cells, number of Ki67 positive cells (nuclei) normalized to the total number of DAPI. Error bars represent SEM. (n=4 mice/group) *p<0.05.

Figure S7. mRNA expression of *Fn1* and *Col1a1* in epididymal and inguinal fat pads on HFD. (a) *Fn1* and *Col1a1* mRNA levels in epididymal fat pad on chow and HFD of *F13a1+/+* mice. **(b)** *Fn1* and *Col1a1* mRNA levels in inguinal fat pad on chow and HFD of *F13a1+/+* mice. **(c)** *Fn1* and *Col1a1* mRNA levels in epididymal fat pad on chow and HFD of *F13a1-/-* mice. **(d)** *Fn1* and *Col1a1* mRNA levels in inguinal fat pad on chow and HFD of *F13a1-/-* mice. **(e)** *Fn1*

mRNA levels in epididymal fat pad of *F13a1+/+* and *F13a1-/-* mice on HFD. **(f)** *Col1a1* mRNA levels in epididymal fat pad of *F13a1+/+* and *F13a1-/-* mice on HFD. **(g)** *Fn1* mRNA levels in inguinal fat pad of *F13a1+/+* and *F13a1-/-* mice on HFD. **(h)** *Col1a1* mRNA levels in inguinal fat pad of *F13a1+/+* and *F13a1-/-* mice on HFD. All error bars represent SEM; (n=4 mice/group); *p<0.05.

Figure S8. The collagen content of skeletal muscle was not affected in *F13a1-/-* mice on HFD. Hydroxyproline assay of total protein extracts from skeletal muscle of *F13a1-/-* and *F13a1+/+* mice on HFD. (n=4 mice/group). Error bars represent SEM; N.S-Not Significant.
